# Current Advancements in Pectin: Extraction, Properties and Multifunctional Applications

**DOI:** 10.3390/foods11172683

**Published:** 2022-09-02

**Authors:** Vinay Chandel, Deblina Biswas, Swarup Roy, Devina Vaidya, Anil Verma, Anil Gupta

**Affiliations:** 1 School of Bioengineering and Food Technology, Shoolini University, Solan 173229, India; 2 Department of Food Science and Technology, Dr. Yashwant Singh Parmar University of Horticulture & Forestry, Solan 173230, India

**Keywords:** pectin, galacturonic acid, methoxyl content, food, health and pharmaceutical applications, food packaging

## Abstract

Pectin is a heterogeneous hydrocolloid present in the primary cell wall and middle lamella in all dicotyledonous plants, more commonly in the outer fruit coat or peel as compared to the inner matrix. Presently, citrus fruits and apple fruits are the main sources for commercial extraction of pectin, but ongoing research on pectin extraction from alternate fruit sources and fruit wastes from processing industries will be of great help in waste product reduction and enhancing the production of pectin. Pectin shows multifunctional applications including in the food industry, the health and pharmaceutical sector, and in packaging regimes. Pectin is commonly utilized in the food industry as an additive in foods such as jams, jellies, low calorie foods, stabilizing acidified milk products, thickener and emulsifier. Pectin is widely used in the pharmaceutical industry for the preparation of medicines that reduce blood cholesterol level and cure gastrointestinal disorders, as well as in cancer treatment. Pectin also finds use in numerous other industries, such as in the preparation of edible films and coatings, paper substitutes and foams. Due to these varied uses of pectin in different applications, there is a great necessity to explore other non-conventional sources or modify existing sources to obtain pectin with desired quality attributes to some extent by rational modifications of pectin with chemical and enzymatic treatments.

## 1. Introduction

Pectin is a plant-based hydrocolloid, commonly added as an ingredient in innumerable food products, in the pharmaceutical industry and in other applications, such as for the development of edible films, plasticizers, paper substitutes and foams due to its unique structural and biochemical properties. The term pectin is applied to identify the numerous polymers that vary in neutral sugar content, molecular weight and chemical configuration, as pectin with different functional properties are produced by different plants. The different functional groups and certain structural modifications enable the application of pectin molecules for numerous purposes [1,2,3], mainly because of its easy accessibility, non-toxic properties and cheap cost of production [4]. Pectin is extracted on a large scale from the fruit waste produced in significant amounts by the fruit processing industries, which, if not utilized, end up in landfills, resulting in environmental deterioration due to microbial degradation and emission of greenhouse gasses [5]. This molecule was firstly isolated from the tamarind fruit in year 1790 by Louis Nicolas Vauquelin. The term pectin was coined from the greek word “pektikos”, meaning to solidify or congeal, in the year 1825 by Henri Braconnot [6]. Pectin represents a family of complex polysaccharides, making up the highest percentage composition of plant mass of approximately 35% in dicotyledonous plant cells, while grasses contain 2–10% [7], and woody tissues contain 5% [8]. Pectin synthesis takes place in the golgi system from UDP-D-galacturonic acid during the initial stages of growth in the enlarging cell walls of plants [9]. Pectin are polymerized in the cis-golgi, methyl esterified in the medial-golgi, while substitution with side chains occurs in the trans-golgi cisternae [10,11]. The development stage of the plant has great influence on the composition and structure of pectin. Pectin acts as the main cementing agent, covalently linking cellulose fibrils to other polymers, and also the intracellular pectin provides a channel for the movement of nutrients and water. These collectively, together with other components of cell wall, help in maintaining the turgor pressure of cell walls, thus determining the growth and extension of plants [1,12]. The pectin carboxyl groups are highly methylesterified when synthesized, but later on, with the onset of maturity, these esters are depolymerized by the pectin methyl esterase enzyme. The pectic substances have been classified into four different types, as (a) protopectin, (b) pectic acid, (c) pectinic acid and (d) pectin by the American chemical society. Protopectin is the parent substance, which is relatively insoluble with no gel-forming properties. Protopectin is commonly present in the inner tissues of plant cell walls [13]. Pectic acid is formed from pectin by enzymes that hydrolyze a methyl ester group from the pectin molecule, and it has no gelling power. Protopectin is the water-insoluble precursor of pectin found in plant tissues, and is converted to water-soluble pectin by restricted depolymerization. The term protopectinase (PPase) was applied to the enzyme that hydrolyses or dissolves protopectin, liberating water-soluble pectin with the resultant separation of the plant cells from each other [14]. Pectic acids contain fewer methoxyl groups, and are water-soluble pectic substances (galacturonans). Pectic acid salts are also known as pectates. Pectinic acids consist of long polygalacturonans with <75% methylated galacturonate units, and the salts of pectinic acids are named as pectinates. Pectin or pectinic acid are soluble forms of pectin polymers, and these are required for gel formation. In pectin (or) polymethyl galacturonate polysaccharide, the carboxyl groups are about 75% esterified with methanol [13].

The pectin oligosaccharides also play a role in enhancing the growth and activity of beneficial gut bacteria, thus playing the role of a potential prebiotic [15]. Pectin possesses good physiological properties such as textural, gel forming, rheological properties, etc. [16], The pectin and pectin-derived compounds also possess various functional properties; for instance, their antioxidant and anti-inflammatory properties [17], antibacterial and antiglycation capacity [18], fat absorption and ion binding capacity [19], production of edible food coatings, treatment of cancer [20] and wound healing and dressing ability [21] have been recently explored by the pharmaceutical industry.

There are already a number of literature review reports on pectin available, such as the extraction of pectin, pectin’s chemistry and pharmaceutical uses, nutraceutical and functional properties, food packaging application of pectin, etc. [2,22,23,24,25]. Nevertheless, there is a limited comprehensive review on pectin covering pectin’s sources, extraction and potential use. Therefore, the current paper reviews the structure and functionality of pectin, pectin extraction methods, the multidisciplinary role of pectin in different fields such as food, packaging, antioxidant and metal binding, and the health enhancing properties of pectin, over the past years.

## 2. Basic Structure of Pectin

Pectin is carbohydrate polysaccharide which possesses many structures with a common saccharide as its subunit. The pectin chains are predominantly of three types [26], primarily consisting of rings of methylated D-galacturonic acid units. The structure of pectin is affected by isolation conditions, storage and processing of plant materials [27]. The D-galactose on oxidation forms D-galacturonic acid, in which the sixth carbon in the R group external to the saccharide ring is transformed into a carboxylic acid group from an alcohol through the process of oxidation [28]. The structure and rigidity of the plant cells is due to the acidic nature of D-galacturonic acid, which provides the charge required for strong intermolecular bonding [8]. The α 1–4 bonding occurs in pectin between the equatorial OH group on the first carbon in the ring of one galacturonic acid and an equatorial OH group on the fourth carbon in ring of a second galacturonic acid. A linear polysaccharide is formed, as the two OH groups involved in bonding are equatorial. Pectin consists of long chains of α-D-galacturonate units joined by α (1 → 4) linkages and 2–3% of l-rhamnose units in association with the galacturonate units joined through β (1 → 2) and (1 → 4) linkages forming the primary chain of pectic substances.

## 3. Chains of Pectin

The chains of pectin are generally classified on the basis of their degree and type of substitution into three types, namely homogalacturonan, rhamnogalacturonan I and rhamnogalacturonan II. Homogalacturonan is a simple chain of polygalacturonic acid, with no substitution, frequently referred to as “smooth” chains; these account for about 60% of all pectins in the cell walls [29]. Rhamnogalacturonan I (RG-I), structurally characterized as a long backbone sequence of alternating galacturonic acid units and D-rhamnose, comprising of spatially regulated polymers [26]. Xylogalacturonan (XG) consists of the galacturonic acid in the backbone attached by xylose in branches, while rhamnogalacturonan II consists of a homogalacturonan backbone substituted with a large variety of complex glycan side chains, containing numerous ranges of neutral sugars. The 1,4 glycosidic linkages are present along the entire homogalacturonan pectin chains, whereas rhamnogalacturonan I is linked through 1,2 glycosidic linkages (C_1_ on the galacturonic acid, C_2_ on the rhamnose), and rhamnogalacturonan II has 1,4 glycosidic linkages (C_1_ on the rhamnose, C_4_ on the galacturonic acid) [26]. Except for rhamnogalacturonan II, these galacturonans do not have a stable definite structure [30]. The helical shape is formed due to the pattern of linking of the chains. Previously, it was considered that pectin molecules consist of homogalacturonan chains as the primary backbone, and rhamnogalacturonan I and II chains inserted as side chains. However, now it is considered that rhamnogalacturonan I may be the primary backbone and homogalacturonan and rhamnogalacturonan II are the major side chains [26].

**Figure 1:**
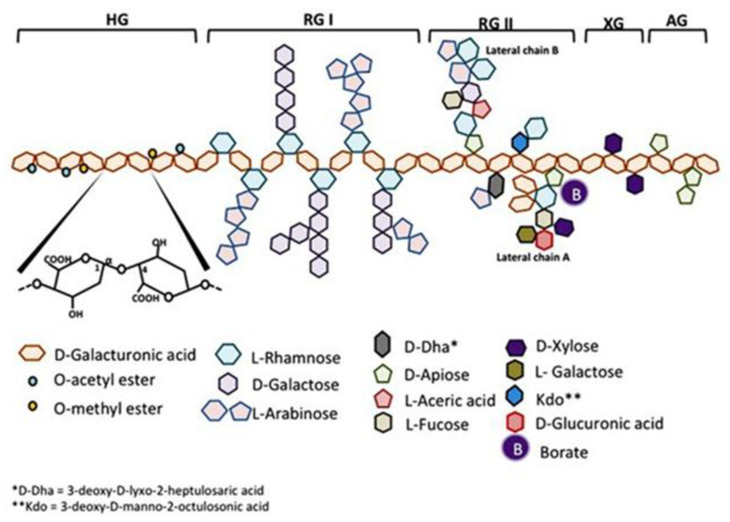
Schematic diagram of pectin structure [34]. Here, HG and AG refers to homogalacturonan and arabinogalactan.

Apart from side chains on RG-I and RG-II, pectin molecules also have a definite amount of acetylation and methyl-esterification on the galacturonic acid units. The hydrogen on the OH group of the carboxylic acid is replaced by a methyl group (CH_3_) during the methyl esterification of the pectin, changing the R group from COOH to COOCH_3_. The depolymerization of the pectin molecule by the application of different methods leads to the production of pectin-derived compounds consisting of a mixture of substituted and unsubstituted polymer fragments with diverse degrees of polymerization [15]. The most frequently applied techniques include enzymatic [31] and chemical [32] pectin modifications. The undesired structural modifications during enzymatic processes can be generally avoided, as enzyme action on substrates is specific due to their precise mode of action, thereby producing specific tailored pectin derived compounds [33]. The structural modification of pectin occurs due to the insufficient action of the pectin-related enzymes to modify the pectin molecule to a definite extent [18]. The schematic representation of pectin structure is illustrated in Figure 1.

## 4. Types of Pectin

Pectin is classified as high methoxyl or high ester pectin when the degree of esterification greater than 50%; however, if the degree of esterification is less than 50%, then it is termed as low ester or low methoxyl pectin [1,12]. The percentage of the amount of carboxyl groups of D-galacturonic acid which have passed through the process of esterification with ethyl alcohol is called the degree of esterification. The particular arrangement of the structure of pectin is based on the esterification of galacturonic acid residues with methyl groups at C-6 and acetyl groups at O-2 and O-3 on the continuous polygalacturonic acid chain of homogalacturonan [35]. The de-esterification of high methoxyl pectin in an acidic environment or enzymatic treatment by pectin methylesterase leads to the production of low methoxyl pectin [36]. The amidated pectin is not produced naturally by plants like high methoxyl and low methoxyl pectin, but can be produced industrially by modification of some of the non-esterified carboxyl groups into amide groups. 

Amidated pectin is synthesized through the reaction of ammonia with carboxymethyl groups (–COOCH_3_) on the pectin molecule [31,32]. The degree of amidation (DA) is defined as the percentage of carboxylic acid groups of pectin present in amide form. The replacement of methoxyl groups with amide groups modifies some properties of the pectin gels; for instance, amidation increases the water solubility of pectins [32] and enables it to be more thermoreversible and withstand more calcium variation [31,37,38]. The replacement of the methoxyl groups of pectins with primary aliphatic amines leads to the production of N-alkyl-amidated pectin conjugates with different functional attributes. Research has been conducted regarding the gelation of LMP and partially amidated LMP (ALMP) induced with Ca^2+^ and high pH [39]. The results revealed that the storage shear modulus (G′) at 1 Hz frequency strongly increased below a characteristic temperature, and crossed the loss modulus (G″) at a characteristic temperature Tc in the presence of Ca^2+^ at pH 6. The gelation of both LMP and ALMP over a series of Ca^2+^ concentrations ([Ca^2+^]) was adjudged as a universal function of T-Tc, and with the increase in [Ca^2+^] concentration, an increase in Tc was observed. The amidated galacturonic pectin plays a comparable role in the complexation of Ca^2+^ ions as the charged galacturonic residues, thus little variation was observed in the gelation of LMP and ALMP.

The gels of high methoxyl pectins are formed in the presence of soluble solids at higher concentration, water and medium pH < 3.5. The stability of high methoxyl pectin gels is due to the formation of intermolecular hydrogen bonds and hydrophobic bonds between methyl esters. These find application in the preparation of jams, jellies, marmalades, sweets and desserts. The low methoxyl pectin forms gels in the presence of divalent calcium ions or multivalent cations at proper concentrations; for example, Ca^2+^ ions over a 2.0–6.0 pH range [1,4,12,40] due to ionic interactions between free carboxyl groups in galacturonic acid and divalent or polyvalent ions [41]. They are generally used in enhancing the stability and texture of water-soluble soy extract, as well as dietary and dairy products [40]. The degree of esterification of pectin is mainly used to establish the gelation and emulsion properties, and it can be detected by use of chromatography, mass spectrometry, Fourier transform infrared spectroscopy (FTIR), FT-RAMAN and other spectroscopy methods [26]. Microarray technology is also used nowadays for estimation of the degree of esterification, and is similar to the technology used in the study of proteins and nucleotides [42]. The components of pectin microarrays include a set of target molecules (pectins), a set of probes to detect the targets (monoclonal antibodies, mAb) and a surface onto which the two compounds are immobilized (slides or membranes). The pectins are immobilized onto a surface and then exposed to probes in a solution. Then, detection of binding of the probes to arrayed pectins is done by fluorescent or colorimetric tags, usually coupled to a secondary antibody. Microarrays constructed using relatively pure and well characterized pectin samples (glycan arrays) are applicable to analyze interactions between pure pectins and ligands or enzymes, where detailed knowledge of defined structural features such as degree of esterification or side chain composition is required [42]. 

Another detection method that is showing promise uses atomic force microscopy [43]. The degree of branching and the structure of pectin can be observed by studying the images obtained by atomic force microscopy analysis of isolated polymers. The structures of pectin isolated from unripe tomato were observed using atomic force microscopy, which led to the conclusion that complex biopolymer consists of HGs held together by RG-I regions [44]. The atomic force microscopy analysis revealed that sugar beet tissue pectin composed of largely un-aggregated chains, among which polysaccharide–protein complexes were 67% composed of a single protein molecule attached at one end of the polysaccharide chains, and 33% were extended stiff polysaccharide chains [45]. Peaucelle et al. (2011) [46], by employing atomic force microscopy, showed that pectin demethylesterification affected the mechanical properties of meristematic cell walls by triggering subepidermal tissue layers, contributing to an increase in the elasticity of these layers, opposite from the results obtained from non-meristematic stem tissue. These different results were apparently because of the different actions of PMEs in different types of cell wall.

The raw material and precipitating agent also greatly affect the degree of esterification. The application of pectin in the food industry is largely dependent on the degree of esterification; indeed, it should be more than 60% as it is influencing the rate of gel formation and gel quality. The intermediate moisture foods are prepared by the standard procedure of incorporating hot ingredients and then being left to solidify by cooling; the high gelation temperature is required for the proper gel formation by pectin with a high degree of esterification [27]. The long extraction time causes the degradation of methyl ester groups present in pectin into carboxyl acid, and ends in the production of pectin with a lower degree of esterification [47]. The conversion of pectin into the protopectin during the maturation leads to the increase in sugars, and thus makes the fruit softer due to the chemical reactions at excessively low pH and high temperatures, which results in the decrease in the degree of esterification [14].

## 5. Sources of Pectin

The presence of a large amount of pectin in fruit and vegetable sources is not the only parameter regarding the use of them as a commercial source of pectin extraction [48]. In the plant cell wall, the concentration of pectin decreases gradually from the primary cell wall towards the plasma membrane, with the highest concentration of pectin located in the middle lamella. The main sources of lucrative extraction of pectin include citrus fruits, apple and the by-products generated after their processing, including citrus peel and apple pomace [49]; alternatively, other sources of pectin comprise of cocoa husk [50], sunflower heads [51], sugar beet [52], pumpkin [53], watermelon [54], pears [55] and potato pulp [56]. Apple pomace contains about 10–15% and citrus peel contains about 20–30% pectin content on a dry matter basis, while in sunflower head residues and sugar beet, about 10–20% pectin is present on dry weight basis [57]. The pectin content reported for non-conventional sources including fruits and vegetables such as apricot, cherries, orange and carrots are 1%, 0.4%, 0.5–3.5% and 1.4%, respectively, on a fresh weight basis [58]. The waste by-products obtained from industries are promising sources for the extraction of pectin; some examples include sugar beet pulp, amaranth, olive pomace and mango waste [59,60,61]. Yields up to 23% of pectin have been reported from sugar beet, depending on the extraction conditions [62]. The sugar beet pectin contains a high content of acetyl groups and neutral sugars, with lower molecular weight, and due to the proteins covalently bound in the lateral chains, thus results in poor gelling ability as compared to conventional sources such as apple and citrus fruits. Thus, despite easy availability, high pectin recovery and low cost, sugar beet pectin finds negligible use as a texturizer [63]; however, the sugar beet pectins possess excellent emulsifying properties, thus making them superior and valuable as compared to pectin extracted from conventional sources [64]. The sunflower head residues left as a waste product after oil extraction possess high galacturonic acid content and high molecular weight with good gelling properties [65]. The sunflower pectins may include about 11.8 to 14.3% insoluble low methoxyl pectin and 3.3 to 5.0% water-soluble high methoxyl pectin [66]. Pectin extraction has also been reported from potato pulp [67] generated as waste by-product from the potato starch industry, pumpkin pulp [68], peach pulp [69] as a residue from juice industry and linseed seeds [70], and have all exhibited significant yields and properties. Apart from yield from pectin extraction, the recent applications and new properties of pectin discovered after research also determine the application and production of pectin on a commercial basis. The rapid increase in demand of pectin due to its non-toxic nature and wide applications in different industries has aroused the necessity for the search of new sources of pectin. However, complete characterization of pectins will be useful to understand the applications we currently can only presume. The sources of pectin and its extraction process is illustrated in Table 1.

## 6. Extraction of Pectin

The pectin extraction from natural sources is a time-consuming and tedious process as the raw material required for pectin extraction, such as fruit peels or pomace, are high in moisture content and as such are prone to breakdown due to the action of fungal enzymes. The pectic enzymes produced by fungi, including the de-esterifying (pectin methylesterase) and depolymerizing (pectin lyase, polygalacturonase and pectate lysase) enzymes, are responsible for the disintegration of pectin. Pectin extraction from apple pomace is more difficult as compared to citrus peels, as it is prone to spoilage by pectolytic enzymes unless instantly dried to reduce the moisture content before further storage for the process of pectin extraction. In some apple varieties, enzymatic treatment of apple pulp is necessary for the efficient extraction of apple juice, thus rendering the apple pomace unsuitable for pectin extraction [89]. The extraction parameters such as particle size, pH, temperature, extraction time and type of extraction solvents greatly affects the yield of pectin [90] and drying methods [91]. The particle size of raw material affects the pectin yield, as in small particles of substrate more protopectin is available as compared to large particles [75,92].

Pectin is commonly extracted from raw materials by aqueous extraction; the most common methods include direct boiling, microwave heating, [72], ultrasonic [73,86], autoclave [93] and electromagnetic induction [71]. The degradation of the quality of pectin to some extent is caused by all of these methods of pectin extraction. The yield of pectin varies with respect to the extraction conditions such as temperature, extraction time, pH and the raw material [94]. The pectin is extracted with mineral acids such as nitric, hydrochloric or sulfuric acid, phosphoric acid and citric acid in an acidic aqueous medium [50]. On an industrial scale, acid extraction and alcoholic precipitation are generally used to extract pectin on a commercial basis. Acid extraction of pectin is based on the fact that hydrolysis of protopectin occurs at higher temperatures [1,95]. The main advantages of using strong acids for pectin extraction are that it provides a high pectin yield and reduces the extraction time, but its disadvantage is that it leads to severe environmental problems such as the disposal of acidic wastewater, alongside the high expenses of energy and chemicals. The food-grade organic acids such as malic, tartaric and citric acids were used to produce pectin from finely ground lyophilized apple peel as an eco-friendly procedure instead of HCl [41] at 85 °C. The molecular weight and apparent viscosity of pectin extracted with citric acid was greater as compared to other organic acid treatments. The pectins extracted with organic acids were highly methoxylated based on the analysis of the degree of methyl esterification. Pectin was extracted from apple pomace by using organic acids such as citric, tartaric, malic and phosphoric acids, and compared with mineral acids including sulfuric, hydrochloric and nitric acids, with citric acid resulting in the highest yield (13.75%), and was determined to be better than the other acids from the point of view of environment and economic factors [75]. The cocoa husks were utilized for pectin extraction using citric acid or hydrochloric acid as extractant at a pH 2.5 or 4.0, and the citric acid resulted in the highest pectin yield (7.62%) at 95 °C for 3.0 h at pH 2.5 [1:25 (*w*/*v*)]; however, uronic acid content (65.20%) was found highest in pectin extracted with water [1:25 (*w*/*v*)] at 95 °C for 3.0 h [76]. The pectin was extracted from apple pomace with 5% (*w*/*v*) citric acid for different time intervals (30, 50 and 80 min) and temperatures (50, 75 and 100 °C); the highest pectin yield (16.8%) was achieved at higher temperatures (100 °C) and a time interval of 80 min [74]. The chelating agents, when used for pectin extraction, influence the functionality of pectin as traces of residues of chelating agents are left in the extracted pectin [96]. The ultrasound and microwave-assisted extraction methods were utilized for pectin extraction from waste lemon, mandarin and kiwi peel using hydrochloric acid (HCl) and nitric acid (HNO_3_) as the extractants [97]. The effect of microwave-assisted pectin extraction was evaluated at microwave power 360–600 W and at 1, 2 and 3 min irradiation time intervals, and on ultrasound-assisted extraction at the temperatures of 60 and 75 °C and sonication time intervals of 15, 30 and 45 min, on the yield of pectin from the peels. The microwave-assisted extraction was found to be better than ultrasound-assisted extraction as the highest pectin yield was recorded with 17.97% yield at 360 W for 3 min, whereas 17.30% was achieved when using HCl as the solvent in ultrasound-assisted extraction at 75 °C for 45 min. Electromagnetic induction was used for pectin extraction from citrange albedo, and extracted protopectin was further compared with that obtained by the conventional heating method [71]. The pectin was extracted at a reduced time period of 30 min with the electromagnetic induction method, compared to 90 min in the conventional method, with significant influence on the structural properties of the extracted pectin.

A higher recovery of pectin was recorded from enzymatic extraction compared to other conventional extraction methods [68,98]. The enzymes degrade the pectin by selective depolymerisation, thus enzymatic extraction is regarded as an environment friendly technique. The effect of Celluclast 1.5 L (Novozymes, Bagsværd, Denmark) at different enzyme concentrations (0.1 mL/kg, 1.05 mL/kg and 2.0 mL/kg) on the physicochemical characterization of gold kiwifruit pectin was evaluated [85]. The highest pectin yield and viscosity were recorded at the enzyme concentration of 1.05 mL/kg Celluclast 1.5 L (Novozymes, Bagsværd, Denmark). The lower pectin yields were recovered by use of either low or high concentration of enzymes, as greater pectin hydrolysis could have resulted due to the use of a high enzyme concentration, whereas at a low enzyme concentration, the pectin yield was low because insufficient enzyme was available to act on the substrate. The early harvested and main harvested gold kiwifruit was utilized for pectin extraction by three methods (acid, water and enzymatic) [99]. Enzymatic treatment resulted in the highest yield, but the molecular weight of the extracted pectin was the lowest. The controlled enzymatic and chemical modifications were used to produce tailor-made citrus pectin-derived compounds from commercial citrus pectin with similar average molecular weights and different degrees of methyl esterification [100]. The action of enzyme endo-polygalacturonase caused the degradation of citrus pectin, and pectin with an average molecular weight (between 2 and 60 kDa) was extracted; furthermore, non-methylesterified galacturonic acid oligomers with a degree of polymerization 1–5 was observed in the separation and identification of extracted samples. When endo-polygalacturonase and pectin lyase enzymes were used in combination, compounds with molecular weights between 2 and 21 kDa were identified, containing methylesterified and non-methyl esterified polygalacturonans with a degree of polymerization from 1–6. When citrus pectin was modified sequentially by chemical saponification and the action of endo-polygalacturonase, a sample of DM 11% and molecular weight 2.7 kDa, containing pectin oligosaccharides with a degree of polymerization from 1–5, was produced. Compared to acid extraction, the enzymes result in higher pectin yield and smaller mass [98]. The chicory roots and cauliflower pectin were extracted with cellulase enzyme, and it was observed that enzyme efficiently hydrolyzed the cellulose from the cell wall. The pectin isolated from pumpkin by enzymes prepared from *Aspergillus awamori* has exhibited lower gel strength (10 KPa) [68,101].

The pectin is usually precipitated with precipitating agents such as alcohol when the pectin solution is concentrated (2–4%), or with aluminum salt if the solution is dilute (0.3–0.5%). The organic solvents are commercially used for pectin extraction, while salts of polyvalent metals are also used to some extent. The effects of acetone, isopropanol and 50/50 isopropanol–acetone as precipitating agents were investigated on the physicochemical and functional qualities of pectin extracted from *B. aethiopum* fruit at an ambient temperature and natural pH of the fruit (5.2–5.5) [84]. Higher emulsifying activity and a better gel forming properties were exhibited by isopropanol-precipitated pectin as compared to the acetone- and 50/50 isopropanol–acetone-precipitated pectins. The increase in pectin yield by 55–90% was observed after the addition of low molecular weight alcohols at 1–3% concentrations in the acid extraction process [102]. Glycerol, ethylene glycol and diethylene glycol had a better effect than monohydric alcohols. A quantifiable increase in the pectin gel strength has been observed with the addition of alcohols. The process for the recovery of pectin from peels of galgal (*Citrus pseudolimon* Tan.) was standardized, and it was found that ethanol precipitation resulted in better quality of pectin than precipitation by aluminum chloride [103]. When precipitation is performed with aluminum salts, aluminum pectinate is produced, which is further washed with alcohol to acidify the aluminum pectinate, then further neutralized with alcohol with slight basicity. The aluminum salt leads to the flocculation of pectin, and it rises to the top, making it easier to skim the products.

### Challenges in Pectin Extraction at a Commercial Scale

Pectin is a hydrocolloid, known for its diverse and unique structural and biochemical properties. The investigations conducted in the past have revealed the multifunctional applications of pectin in different fields, particularly due to some alterations in the structure and branching pattern of chains of pectin. The modifications in the chains of pectin can be induced by changes in the extraction conditions, and also by the source utilized for the pectin extraction. More recently, the main focus has been on the development of environmentally sustainable production of pectin at a commercial scale. The main bottlenecks related to the extraction of pectin are that this process requires the blanching and drying of raw material, a huge amount of water, collection and utilization of the source of pectin (most commonly the waste left by fruit and vegetable processing industries), chemicals/enzymes which play an important role in creating the optimum conditions for pectin extraction, excessive amounts of precipitants for the precipitation of pectin (1.2 to 9.9 L for the production of 10 g of pectin) and the consumption of large amounts of energy by equipment such as mechanical grinding, freeze-drying and heating equipment, etc. [104]. In the past, pectin was extracted mostly by acidic hot extraction at a temperature of 80–100 °C, with long extraction times (30–180 min), using chemicals such as hydrochloric acid, nitric acid or sulfuric acid, which were corrosive, unsafe for use and had high environmental impacts, along with low pectin yields [75,92]. Due to the above-mentioned drawbacks in the conventional pectin extraction process, new protocols have been developed with some alterations of the old conventional methods to enhance the quality and yield of pectin. 

The green extraction protocols and new methods such as ultrasound- or microwave-assisted techniques with reduced energy and reagent consumption, shorter extraction time and greater safety are replacing the old methods, as these are promising options for the sustainable extraction of pectin, with reduced processing time and exploiting environmentally friendly extractants. However, future research is still required for new extraction methods for efficient, reliable, economic, reproducible and environmentally safe extraction of pectin. The deep eutectic solvents (DESs), ecofriendly acids such as citric acid recovered from organic sources or produced by fungi such as *Aspergillus niger*, can be used instead of conventional mineral acid to reduce the pH required for effective extraction; indeed, these perspectives can further promote the sustainability of the pectin extraction process. 

## 7. Interactions of Pectin

### 7.1. Solubility and Dispersibility

The pectin hydrocolloids are commonly identified as water-, chelator- and diluted alkali-soluble pectin on the basis of the solvent. The water-soluble pectin contains a fraction of molecules which are non-ionic in nature and are bound non-covalently to the plant cell walls [105]. The chelator-soluble pectin fraction can be extracted using imidazole, and the pectin polymers are linked by ionic bonds to the cell wall, e.g., via calcium ions [106]. The pectin polysaccharides are bound by covalent ester bonds to the cell wall in diluted alkali-soluble pectin fractions [105]. Greater solubility is exhibited at a higher degree of methylations; however, it decreases with the increase in polymer size [30]. The pectin degradation and change in solubility due to β-elimination reactions are more predominant under weak acidic and neutral conditions, along with an increase in temperature. The β-elimination reaction causes the cleavage of the glycosidic bond located at C-4 and the removal of the hydrogen atom at C-5 of the galacturonic acid unit [107], resulting in the formation of a double bond. The β-elimination reaction occurs more frequently with an increase in pH and temperature, a high degree of methylation and the presence of monovalent salts and EDTA [30].

### 7.2. Gelation

Gelation can be simply defined as the process that involves partial dissolution and partial precipitation of solutions [108]. The structure and properties of pectin such as gelling ability and viscosity are greatly influenced by the source and method of extraction [57]. The gel formation by pectin depends on factors such as the structure of pectin, sugar concentrations, pH, temperature and the presence of crosslinking agents. The gels are physically characterized as the consequence of the formation of a three-dimensional network of cross-linked polymer molecules holding water and solute molecules [26]. The bonding qualities of high methoxyl pectin are used to a great extent in the preparation of products such as candy and intermediate moisture foods that have long-term stability, even at high temperature environments. This is mainly due to hydrogen bonding, and hydrophobic forces play a significant role in gel formation [26,108], both of which are temperature independent. High methoxyl pectin forms gel at high sugar concentrations (>55%) and in acidic conditions with pH < 3.5 [57]. Under the acidic conditions, the electrostatic repulsive forces are weak between the pectin chains, due to the decrease in the dissociation of carboxyl groups of galacturonic acid units. The hydration of pectin molecules is greatly influenced by sugar concentration as the hydration of pectin molecules is reduced with the increase in sugar concentration. The mechanism of gel formation in low methoxyl and amidated pectins is different from high methoxyl pectins as hydrogen bonding is absent, and the intermolecular bonding occurs through the formation of dimmers, using divalent cations, such as Ca^2+^, Fe^2+^ and Zn^2+^, etc., thus forming cross-linking, using two carboxylic groups known as the egg-box model [109]. The low methoxyl pectin forms gels at a higher pH in the range of 3 to 7, and furthermore, the addition of sugar is not essential for gel formation [110].

### 7.3. Breakdown of Pectin

Pectin polymers are highly methyl esterified; consequently, the structure and properties of pectin can be modified by fungal pectinolytic enzymes such as pectinmethyleserases, polygalacturonases and lyases [111]. These enzymes are responsible for causing significant changes in the structure of plant cell walls, such as loss of firmness, reduced shelf-life and quality characteristics. The pectin methylesterases catalyze the demethyl-esterification, thus causing hydrolysis of the methyl-ester group at the C-6 carboxyl, thereby releasing methanol. Polygalacturonase is commonly found in two forms, endo-PG and exo-PG, which can both catalyse the hydrolytic cleavage of the α-(1,4) glycosidic linkages. Pectin lyases (PL), by catalyzing β-elimination, degrade glycosidic bonds without introducing water across the oxygen bridge. β-galactosidase (β-Gal) are responsible for removing galactosyl, and α-L-arabinofuranosidase (α-L-Af) for removing arabinosyl residues from cell wall polysaccharides [111,112]. The degree of haziness and cloudiness during the pressing of the fruits for juice and concentrate preparation in industry is regulated by the application of pectinolytic enzymes to enhance the juice yield.

## 8. Potential Applications of Pectin

**Figure 2:**
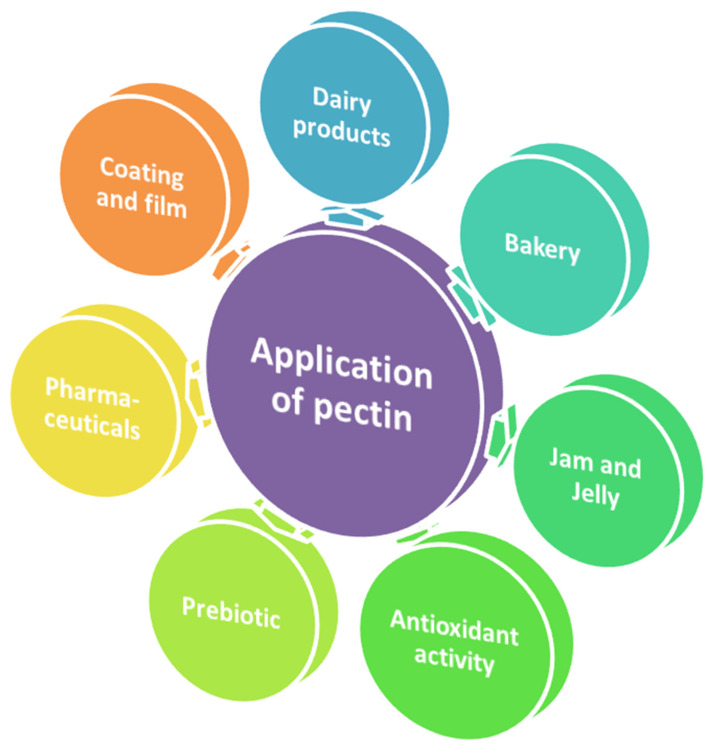
Multifunctional applications of pectin.

The application possibilities of pectin are very wide and numerous, ranging from the major categories of food applications, and the industrial and pharmaceutical sectors (Figure 2). In this review, the application of pectin is primarily classified into three types: food sector, health and pharmacy, and food packaging; all of them are briefly presented in Table 2.

### 8.1. Food Industry

#### 8.1.1. Jams, Jellies and Emulsifying Agent

Pectin hydrocolloids are the main ingredient for the preparation intermediate moisture foods, such as jellies, jams, marmalades, confectionery, pastries and yogurts, etc., due to their versatile gelling properties [105]. Pectin is added late in the process, subjecting it to less heating and leading to convenient and complete dissolution. High methoxyl pectin is used as the gelling and texturizer agent in jams and jellies [143]. The trend for products without or with low sugar, such as dietetic foods, low-calorie jams and carbonated beverages, particularly due to calorie conscious consumers and to fill the need for sugar-free products for diabetics, has led to an increase in demand of low methoxyl pectin. A marmalade with maltitol and amidated citrus pectin (low methoxyl pectin) was developed for these types of consumers without many differences in the characteristics compared to commercial marmalade [114]. The oil/water emulsions can be prepared from sugar beet pectin, which makes poor gels, but works very well as an emulsifying agent [144]. The sugar beet pectin acts by coating the lipid molecules, thus protecting the hydrophobic lipid molecules from water and preventing them from clumping to stay away from water molecules [144].

#### 8.1.2. Bakery Products

Pectin has also received attention from bakers due to its properties, and it can be used as a structure-forming agent, an oxidizing effect enhancer in the dough production process and as a sorbent for improving the quality of bakery products [117,145]. The consumption of foods enriched with pectic substances is becoming an essential part of our diet as these products boost the body’s resistance to adverse environmental conditions, and also due to their ability to bind toxins introduced through food or formed in the body [146,147]. It was established that pectin promotes the fermentation, biochemical and microbiological processes in the dough, and also affects gluten springiness. In the production of bread, pectin has been observed to facilitate an increase in dough volume by holding gas in the dough, retaining structure and hindering the staling process [148]. The increase in the volume of bread up to 13% was exhibited when pectin was added to the composite flour of wheat, maize and cassava [149]. The pectin hydrocolloids improve bread volume primarily by increasing moisture retention and enhancing the viscoelastic properties of dough. The addition of high methoxyl pectin to the wheat bread led to the preparation of bread with a better specific volume, a softer crumb and improved moisture retention qualities [150].

#### 8.1.3. Prebiotic Properties

Pectin and pectin oligosaccharides, consisting of galacturonic acid (GalA), rhamnose (Rha), arabinose (Ara), and galactose (Gal), have been used as prebiotic substrates, as these compounds stimulate the activity and growth of beneficial gut bacteria [15]. The pectin administration to a simulated gastrointestinal tract resulted in a 1000-, 100–1000- and 10-foldincrease in the number of bifidobacteria, bacteroids and faecalobacteria, respectively [151]. Pectin-derived oligosaccharides are manufactured by the depolymerization of purified pectin or by partial enzymatic hydrolysis of raw materials [152]. Pectin-derived oligosaccharides have shown properties of stimulation as these enhanced the growth of beneficial bacteria in the colon, apoptosis of colon cancer cells, and protection against various pathogens [152]. Pectin and dietary fibers provide diverse health benefits, including improvement of physical bowel function, slow gastric emptying, reduced glucose and cholesterol absorption and the increase of fecal mass. Pectin is not affected by salivary enzymes or gastric acid, and it is also resistant to pepsin, trypsin and rennet [153]. The pectin derived from different sources such as apple [154] or citrus [155] can serve as valuable carbon sources for the growth and development of gut bacteria [156]. 

#### 8.1.4. Stabilizing Acidified Milk Products

Pectin is used as stabilizing agent for acidified milk products in the dairy industry. Low esterified pectin is commonly utilized for low-fat yogurt production, as it decreases syneresis due to interaction with milk proteins, thus resulting in a homogenous texture [119]. Conversely, high-esterified pectin is appropriate for the preparation of acidified dairy drinks [157,158]. At low pH, the milk protein is stabilized due to the electrostatic interactions between the positively charged protein and negatively charged pectin, and the uncharged regions of high methoxyl pectin form entropy rich loops, thus repelling the proteins [120]. The pectin addition enhanced the organoleptic acceptance of yogurt, as it increased the viscosity and entrapped volatile compounds inside the matrix. Pectin possess a good water holding capacity, which leads to a decrease in the syneresis of the yogurt [159]. The aggregation of casein results in the sedimentation of the milk particles. The casein particles electrostatically bind to the homogalacturonan domains of pectin, thus preventing clumping and sedimentation of dairy products. The formation of a set yoghurt matrix occurs because anionic interactions such as Ca^2+^ are sensitive to low methoxyl pectin, which promotes interpenetration of hydrated pectin chains into the protein network and steric stabilization, where pectin adsorbs to the surface of casein aggregates through its carboxyl groups [158]. The interaction of pectin cations, mainly divalent minerals such as calcium, is similar to the impact of calcium on probiotic bacteria, resulting in the adhesion to epithelial cells owing to the role of calcium as a bridge among the epithelium surface and negative charges of microorganisms [160].

### 8.2. Health and Pharmaceuticals

#### 8.2.1. Reduction in LDL Plasma Concentrations

Pectin has been revealed to play an important role in reducing LDL plasma concentrations in humans, as blood LDL cholesterol levels were slightly reduced by 3–7% after consumption of 15 g/day pectin over a period of 4 weeks [129]. Pectin coats the micelles, thus keeping them from clumping together by causing electrostatic repulsion between the micelles, increasing the viscosity in the intestinal tract, resulting in reduced absorption of cholesterol from either bile or food. Pectin helps by preventing bile acids from breaking down ingested fatty acids into the smaller fragments required for digestion and absorption into the intestinal wall. This effect is also related to the source of pectin, as apple and citrus pectin were found to be more effective in reducing LDL plasma concentrations than orange pulp fiber pectin [129]. 

#### 8.2.2. Antioxidant Activity

The antioxidant capacity of pectin is attributed to its capability to chelate metal ions [1], and it is influenced by the raw material, the method of extraction and the degree of esterification of the pectin. The application of synthetic additives can be reduced, and clean label products can be produced by the incorporation of pectin to food emulsions as an antioxidant [130,131]. Celus et al. investigated the modification of citrus pectin and evaluated the role of the degree of esterification and oxidative stability of flaxseed/sunflower emulsions. The degree of esterification was appreciably influenced by the oxidative stability of the emulsions, and the low methoxyl pectin (DE ≤ 33%) displayed greater lipid antioxidant potential than the high methoxyl pectin with (DE ≥ 58%); thus, low methoxyl pectin can be successfully used as a natural substitute to synthetic antioxidants [130].

#### 8.2.3. Therapeutic and Pharmaceutical Uses

Pectin is used as an effective remedy for constipation, such as low intestinal motility, dry stool and painful defecation, as it absorbs water molecules in the intestine; this occurs as hydrogen bonds and pectin swell in the media, which leads to an increase in the volume of the water content [161]. The gastric-perfuse pectin resulted in the increased water-holding capacity of feces in mice as the water content increased up to 26%, which induced anti-constipation in vivo [138]. 

Pectin hydrocolloids are also used as wound-healing agents, as they absorb wound exudates, thus preventing excess liquid accumulation at the wound site, while simultaneously providing moist media that encourages collagen synthesis and cell differentiation [21]. The healing and cell regeneration process can be controlled by drug or growth factor-loaded pectin [94]; further microbial activity is restricted by pectin dissociation as it leads to the development of an acidic environment [162]. Pectin is more effective in combination with adjuvant bioactive materials; for example, the pectin–honey complex exhibited better healing results in rats compared to pectin alone and the control. The antibacterial character and hygroscopic properties of honey and pectin play a role in providing a low pH, thus creating an occlusive scaffold to facilitate cell epithelialization [21]. The hydrogen interactions between pectin and mucin improved its binding to the surface. This characteristic is constructive when pectin is used in drug delivery systems to lengthen the residence time of the loaded drug in the site and efficiency of treatment. High RG-I branching structure causes enhanced gastric and small intestinal resistance of Ca^2+^-pectin beads, and thus has been applied as carriers for the colon delivery of drugs [163].

Pectin in combination with traditional cures has also been examined in cancer therapy [164]. Galectin-3, a pleiotropic protein, has a carbohydrate binding domain and exists in intra- and extra-cellular sites, and plays relevant biological roles in different diseases, among which fibrosis, cancer and heart diseases deserve a special mention [20,165]. Galectin-3 is overexpressed in cancer cells and the tumor microenvironment, by which an immunosuppressive response is observed. The inhibition of galectin-3, along with immunotherapy, would decrease tumor growth [166]. Modified citrus pectin (commercially named PectaSol-C and GCS-100) has exhibited considerable impact on galectin-3 inhibition, thus reducing metastasis and tumor size and growth. Both formulas had a synergistic impact on the therapeutic medicines commonly used in cancer suppression [167]. The neuroprotective effects of injected modified citrus pectin were observed after aneurysmal subarachnoid hemorrhage in mice, which was related to the downregulation of galectin-3. The carrier pectin can act as a specific route of cytotoxic drug delivery to abnormal cells as it triggers galectin-3 by binding to its carbohydrate-binding domain [142].

#### 8.2.4. Glycemic Control

Pectin has demonstrated numerous health benefits such as reducing blood glucose and cholesterol levels, lower caloric intake due to increased satiety and improved insulin resistance. Pectin has shown beneficial effects in the prevention and treatment of lifestyle diseases such as diabetes and obesity. Soybean seed coats left as a by-product from soybean processing are a good source of pectin and fiber [168]. Two groups of fifteen healthy men were administered control and soy pectin, and blood samples were collected 15 min before feeding and after consumption of control solution with added soy pectin or the control solution, at intervals of 0, 15, 30, 45, 60, 75, 90, 120, and 180 min. The results indicated that compared to the control, the mean glucose for the soy pectin treatment was lowered by 13.2% for 3 h L^−1^. The pectin cannot be broken down by the enzymes excreted by the stomach or pancreas, and it is only acted upon by enzymes in the colon; therefore, fewer food molecules are absorbed into the body and are available to raise glucose levels in the blood. The intake of unripe apple powder rich in pectin was assessed to measure the hypoglycemic activity of healthy volunteers. After feeding, it was observed that glucose metabolism improved by increasing urinary glucose excretion, thus leading to the conclusion that apple pectin can be administered to reduce postprandial glycemia and diabetes [169].

#### 8.2.5. Pectin as an Encapsulating Agent

Pectin can directly bind to food components, forming a protective layer, similar to other biopolymers such as chitosan and alginate which are also used for encapsulation and coating purposes [170]. Pectin alone or in combination with other biopolymers was used for encapsulating purposes in foods to mask the detrimental smell of some bioactive compounds, e.g., fish oil [171], or to protect volatile and sensitive components from harsh environmental conditions [136,172]. Recent research improved the encapsulation efficiency of maltodextrin by forming a complex with high methoxy pectin to coat walnut green husks, and found that pectin combined with maltodextrin resulted in higher antibacterial activity against *Bacillus cereus* and *Staphylococcus aureus* as compared to maltodextrin alone, which might be due to better protection and controlled release of walnut green husk by pectin [172]. 

#### 8.2.6. Metal Binding Properties

Pectin prevents the absorption of metals as it binds with them in the digestive tract [173]. Pectin is generally administered orally to eliminate heavy metals, and reduce lead absorption and strontium levels in bones and blood [174,175]. Specific chelators are used for treatment of toxic metal poisoning; however, they have some side effects such as the relocation of metals in the brain or bones, and a decline in critical minerals, affecting the gastrointestinal functions and occurrence of skin pains [176]. Pectin polysaccharides interact more efficiently with metals than the chelators due to the three-dimensional structure [177]. The mechanism of metal binding by pectin is based on the “egg-box” model; the esterified residues are inactive, while the covalent bonds are formed between negative charges of free carboxyl groups in the pectin molecules and valence metal ions [94].

### 8.3. Food Packaging

Pectin also finds another major application in the food industry for the production of edible coatings and packaging for food preservation due to its ability to form gels and films, considered as green and environment friendly alternatives, important for the development of the sustainable world [1,178,179]. The petroleum-based polymers are commercially used for preparation of food packaging materials and edible coatings; however, they are associated with the fast exhaustion of these natural resources [180]. Pectin coatings are renewable, biodegradable, biocompatible and have been found to extend the shelf-life of food products as compared to petroleum-based polymers [181]. The film and coatings played a significant role in extending the shelf-life by controlling water loss, maintaining firmness and reducing fruit decay [182,183,184]. Edible pectin films and coatings limit the physiological degradation of fruits during storage by altering the surrounding atmosphere and modifying oxygen levels inside the fruits, thus impeding the fruit ripening process and the loss of bioactive compounds [181].

#### 8.3.1. Packaging Film

Pectin possesses the ability to form gels and impart firmness, characteristics which are significantly employed for the formation of films in food packaging. The gelation behavior is dependent on the presence of calcium ions, acids or sugars. These films prevent the spoilage of food by acting as a barrier between the external and internal environment. These pectin-based films serve as a recent advancement in the food industries where the properties of the pectin were modified by incorporating additives in the formation of films to improve the tensile strength and extend the shelf-life of the packaged food [23,185].

Pectin alone is not very effective for the making of films since they are fragile and more hydrophilic in nature with deprived mechanical properties [186]. These properties defy the usage of pectin-based films for food packaging. Hence, the functionalization of the active residues of pectin was accomplished by adding bio-active materials such as essential oils, nanoparticles, free fatty acids and some phenolic compounds that notably enhanced the film characteristics to a greater extent and imparted antimicrobial activity [175,187,188]. Here, pectin will serve as the entrapment matrix where the bioactive compounds were integrated to produce a film. In addition, plasticizers are low molecular weight compounds that are included along with biopolymer casting solution to ameliorate the properties of the film. These plasticizers will help in increasing the interchain distances between polymers and confer good flexibility by reducing firmness and cohesion. Some of the commonly used GRAS (generally regarded as safe) plasticizers include sorbitol, glycerol, sucrose, polyethene glycol (PEG), mannitol, etc. [174,189]. These substances increase the polymer chain flexibility by reducing the deformities, hardness, viscous nature, density and electrostatic charge of the polymer. They also improve the mechanical properties of the film by facilitating the formation of hydrogen bonds between macromolecules, thus reducing the inter-intramolecular interaction [186]. Plasticizers should have a low vapor pressure and low diffusion rate, and should be compatible with the polymer. Usually, blend films are prepared by incorporating pectin with other polysaccharides or protein for various purposes, and are specially employed in food packaging [184,190,191,192,193,194]. The common method of film fabrication involves casting from solvent and extrusion/blending. The casting approach involves complete dispersion of biopolymer and natural additive in an appropriate solvent, followed by pouring, drying and recovery of the film in a petri dish [195]. The extrusion process is scalable and broadly employed in industries where active compounds are mixed with biopolymer inside an extruder, and ultimately the films are obtained by compression, blowing or melting [196]. An edible film based on aqueous emulsions of LMP (1%) with purified sunflower waxes (0.3%) has been prepared; this material exhibited a low vapor transfer rate and good water resistance [197].

Nanoparticles, essential oils (EO) and other bioactive components are widely studied for their antibacterial and antifungal activity against various food-borne pathogenic organisms. Usually, various types of functional bioactive agents are employed in pectin-based active films because they impart functional activity in pectin-based films. Pectin/agar blend films prepared by Roy et al. (2021), with added melanin nanoparticles and grape seed extract, exhibited intense antioxidant and antimicrobial activity against various food pathogens [187]. In another report, pectin-based film was fabricated using zinc sulfide nanoparticles to improve the antibacterial performance of the film [185]. Carvacol and cinnamaldehyde added to pectin films was reported to be very effective in improving the shelf-life of ham and bologna [124]. In another work, lime peel extract and coconut water was included in pectin film and used for soybean oil retardation [122]. Pectin-based film mixed with cinnamon oil was also effective in delaying the growth of microbes in dry tofu during storage [123].

#### 8.3.2. Coating Material

Pectin-based coatings have also received considerable attention recently. The coating can be achieved by using various well-known methods, such as dip coating, spray coating, electro spraying, the panning method, etc. [198]. There are plenty of reports on the use of pectin and functional bioactive compound formulation as coating materials to enhance the shelf-life of fruits, meat and other food products [23,178,186,199]. Recently, pectin-based coating including orange and lemon EO was used to extend the shelf-life of apple slices, and the study showed that the coating decreased the microbial count and weight loss of the apple [128]. In another report, cinnamon oil included in pectin-based formulation, when used in grape coatings, improved the shelf-life by reducing the fungal growth and enhancing the antioxidant activity [200]. Recently, oregano EO and resveratrol incorporated pectin solution was used for pork loin coating [125]. The coating was effective in reducing the lipid oxidation, as well restricting the growth of microbes. Apple pectin-based coating functionalized by lemon EO was used for strawberry storage, and it was reported that the coated strawberry could be stored for a longer time [201]. In a recent work, Moradi et al. studied the effect of lemon and mint EO added to pectin coating solution on rainbow trout. They demonstrated that the coating effectively reduced the oxidation, and helped to maintain the texture and color of the trout [126].

## 9. Conclusions and Future Perspective

Pectin is extensively used as a texturizer, stabilizer and emulsifier in a variety of foods and other applications. The use of pectin as a fat and sugar replacement in low-calorie foods is likely to increase in the future with the rising demand for these foods. The commercial sources for pectin extraction are very limited, apart from its occurrence in a large number of plant species. Therefore, there is a great need to search for other sources or modify the existing sources to attain pectin of desired quality attributes. Pectin is an important component of food and pharmaceutical products due to its gelling and stabilizing property. It is positively effective in wound healing, and has a synergistic effect on medicines in cancer therapy. Pectin is a composite molecule with vast usage, and current knowledge of the molecular basis of gelation has helped us to understand some aspects of this complex phenomenon. Studies regarding pectin biosynthesis pathways in plants are extremely important, as well as research regarding its metabolism in human beings and animals. The market for pectin and pectin-derived hydrocolloids is vast and is projected to grow; pectin application is common in the food and non-food industries alike, with yet more applications being discovered. Therefore, further research on the advancement of methods for detailed analysis of the structure and properties of pectin polysaccharides is crucial.

## Figures and Tables

**Table 1:** Sources of pectin and the extraction methods.

Source	Pectin Yield	Extraction Method	References
Citrange	29% (*w*/*w*) of dried albedos	Acidic extraction with a solution of 1 M H_2_SO_4_ and electromagnetic induction heating	[71]
Lime peel	5.20 to 23.59%	Acidic extraction with hydrochloric or citric acid and microwave and conventional heating methods	[72]
Grapefruit peel	23.44–26.74%	Acidic extraction with 0.5 M HCl using ultrasound-assisted heating extraction	[73]
Apple pomace	5.7–16.8%	Acidic extraction with 5% (*w*/*v*) citric acid	[74]
Apple pomace	13.75–17.82 g % of pectin on a dry basis	Acidic extraction with citric or nitric acids	[75]
Cocoa husk	2.0–9.0%	Acidic extraction with HCl using microwave heating	[50]
Cocoa husks	3.38–7.62%	Acidic extraction with citric acid or hydrochloric acid at pH 2.5 or 4.0	[76]
Sunflower heads	15–25%	Alkaline washing, 16 °C for 25 min at pH 5–7, 28:1 solvent:solid ratio	[51]
Sugar beet	4.37–28.84%	Enzymatic extraction with xylanase, cellulase and their mixtures (1–4 h), and ultrasound-assisted treatments	[77]
Sugar beet (pressed, ensiled and dried pulp)	13–19%	Acid method or a commercial cellulase	[78]
Pumpkin	10.03 and 8.08 g/100 g	Enzymatic extraction by cellulase and α-amylase	[53]
Watermelon	WRP yield (13.4%),	Acidic extraction by 1 M HCl solution	[79]
Watermelon	14.2–19.35%	Acidic extraction with 0.1 M nitric acid for 1 h	[54]
Pears	68.40–42.00 (g ethanol insoluble material/100 g material) dry basis of material processed; 6.84–4.20 (g ethanol insoluble material/100 g material) dry basis of material processed	Enzymatic extraction by a recombinant polygalacturonase	[55]
Potato pulp	14.34–4.08%	Acidic extraction with HCl, H_2_SO_4_, HNO_3_, citric acid and acetic acid	[56]
Sugar beet pulp	6.3% to 23.0%,	Acidic extraction by citric acid	[62,80]
Banana peels	15.89–24.08%	Acidic extraction by citric acid	[81]
Mango peel pectin	13.85%	Acidic extraction at pH 1.5 by 2 M HCl using the microwave-assisted technique	[82]
Linseed seed	0.35–0.65%	Alkaline extraction procedure, 0.1 M HCl at pH 2	[70]
Pomegranate peel pectin	8.5%,	Acidic extraction using 1 M nitric acid.	[83]
Palmyra palm	102.3–105.8 (g kg^−1^)	Acidic extraction using 0.1 mol/L HNO_3_	[84]
Cashew apple pomace	10.7% to 25.3% dried raw material	Acid extraction conditions with 1 N HNO_3_	[35]
Gold kiwifruit pectin	4.00 to 5.40% (*w*/*w*) on dry matter basis	Enzymatic extraction (Celluclast 1.5 L, Cytolase CL, Cellulyve TR 400 and NS33048)	[85]
Pistachio	10.3–12.0%	Acidic extraction using citric acid, hydrochloric acid and sulfuric acid with ultrasound-assisted extraction	[86]
Mangosteen rind pectin	1.16 ± 0.17%	Acidic extraction using H_2_SO_4_ at pH 2	[87]
Artichoke	65.9 ± 2.1 mg/100 mg	Enzyme extraction using Viscozyme^®^ L Novozymes	[88]

**Table 2:** Pectin utility and application potential.

Application Type	Pectin Utility	Key Finding	References
Food Industry			
	**Jams and Jellies**		
	Jams and marmalade from French Plantain peel	French Plantain peel was successfully utilized for jam and marmalade preparation with nice spreadability and overall acceptability.	[113]
	Marmalade for patients with type 2 diabetes	The marmalade was prepared with agar-gelatin, and pectin-based marmalades with maltitol, dried fruits and berries for glycemic control. The marmalade was successfully developed with textural parameters such as ‘‘gumminess,’’ ‘‘springiness,’’ and ‘‘homogeneity”, and organoleptic properties with comparable overall consumer acceptance for both healthy people and people suffering with type 2 diabetes.	[114]
	**Emulsifying agent**		
	Watermelon rind pectin as emulsifying agent	The watermelon rind pectin displayed exceptional emulsification capacity, incorporating up to 60% (*v*/*v*) oil in the emulsions. The emulsions were stable for longer periods because of its protein content acting as surface active materials and the steric repulsions between droplets caused by long chain branches of RG-1 enriched pectin.	[115]
	Pectin-based microgels as emulsions	The pectin-based microgel sensitivity varied with changes in pH and ionic strength and influenced the stability of emulsions. After emulsification, the pH of the emulsions was adjusted from pH 4.2 to 4, 3 or 2, and they remained stable for at least three weeks.	[116]
	**Bakery products**		
	Wheat flour bread	Apple pectin was successfully used for the preparation of wheat flour bread quality with improvement in the activation of fermentation and acid accumulation processes. The bread crust had a thin-walled crumb, with high porosity and sorption capacity.	[117]
	Wheat composite dough and bread	Composite flour of wheat, pearl millet, and Bambara groundnut were used for bread production, along with apple pectin. The pectin exhibited up to 1.5% improved dough stability, whereas the highest overall acceptability for composite bread was observed at 2% pectin addition.	[118]
	**Prebiotic properties and stabilizing acidified milk products**		
	Low fat yoghurt	The low-fat set yoghurt, with enhanced bacterial counts, was prepared with the addition of low methoxyl pectin contributing towards metabolite production, thus accountable for the higher acidity and antioxidant potential. This resulted in enhanced physico-chemical quality, rheology (elastic, viscous modulus or complex viscosity) and sensory liking.	[119]
	Carboxymethylcellulose and pectin effect on the stability of acidified milk drinks	The acidified skim milk drinks were not stabilised after a period of time, but the whole milk drinks exhibited a noticeably reduced formation of serum phase after the addition of the combination of high methoxyl pectin (HMP) and carboxymethylcellulose (CMC) polysaccharides. The drink stability was enhanced when the amount of HMP increased in the polysaccharide ratio.	[120]
	Sugar beet pulp pectin and lemon peel waste prebiotic potential	Sugar beet pulp and lemon peel waste pectic oligosaccharides have prebiotic properties; joint populations of bifidobacteria and lactobacilli increased from 19% to 29%, 34% and 32% in cultures, respectively.	[121]
**Packaging industry**			
	**Food packaging film**		
	Pectin/lime peel extract/coconut water-based film	Pectin-based functionalized film was useful in the retardation of vegetable oil during storage.	[122]
	Pectin/cinnamon oil film	The film, when used for tofu storage, showed enhancement in shelf-life by reducing the growth of unwanted microbes.	[123]
	Pectin/Carvacrol/Cinnamaldehyde film	The pectin-based film reduced the growth Listeria in the ham and bologna, and it shows a better response towards ham.	[124]
	Pectin/carbon quantum dot film	The film showed excellent antioxidant activity and good antimicrobial potential, which could be useful for food packaging applications.	
	**Food coating**		
	Pectin/oregano oil/ resveratrol	The pectin formulation-coated pork loin showed less lipid oxidation and low microbial growth compared to uncoated counterparts.	[125]
	Pectin/ lemon EO/mint EO	The rainbow trout coated with pectin-based solution preserved the texture and color, as well as delayed the oxidation.	[126]
	Pectin/eugenol	The melon coated with functionalized pectin solution reduced the growth of Listeria while in storage.	[127]
	Pectin/lemon EO/orange EO	The formulation-coated apple slice showed lowered microbial count and less weight loss compared to the untreated sample.	[128]
**Health and Pharmaceutical industry**			
	**Reduction in LDL plasma concentrations**		
	Cholesterol-lowering properties of different pectin types	The trials revealed that a high degree of esterification and high molecular weight pectin were important for cholesterol lowering in mildly hyper-cholesterolemic persons. In a successive 3-week trial with 6 g/day pectin, citrus DE-70 and high MW pectin DE-70 reduced low-density lipoprotein by 6–7% as compared to the control (without changes in total cholesterol).	[129]
	**Antioxidant activity**		
	Mangosteen pectin antioxidant activity	The mangosteen pectin showed antioxidant activity with an IC_50_ of about 161.94 ± 31.57 ppm.	[87]
	Structurally modified pectin for lipid antioxidant capacity in linseed/sunflower oil-in-water emulsions	The citrus pectin 5% (*w*/*v*) was added in linseed/sunflower oil emulsions stabilized with 0.5% (*w*/*v*) Tween 80, and examined during two weeks of storage at 35 °C. The higher antioxidant capacity was observed in low demethylesterified pectin (≤33%) than high demethylesterified pectin (≥58%), probably due to its higher chelating capacity of pro-oxidative metal ions (Fe^2+^), whereas the arrangement of methylesters along the pectin chain slightly affected the antioxidant capacity.	[130]
	Antioxidant activity of pectin from hawthorn wine pomace	The antioxidant activity was evaluated for hawthorn wine pomace pectin extracted by different methods by using the concentration (IC_50_) index, DPPH scavenging ability, and the IC_50_ values were 0.01 (VC, ascorbic acid), 2.63 (hydrochloric acid method), 2.10 (citric acid method), 2.24 (cellulase method) and 3.11 (microwave-assisted chelating agent method) mg/mL.	[131]
	**Metal binding properties**		
	Pectin-based aerogels properties for adsorption of Pb^2+^	Novel porous pectin-based aerogels (PPEAs), prepared by incorporating polyethylenimine (PEI) using ethylene glycol diglycidyl ether (EGDE) as a cross-linker, have several desirable features, such as a maximum Pb^2+^ adsorption capacity (373.7 mg/g, tested at pH 5.0), are ultralight (as low as 63.4 mg/cm^3^), with high mechanical strength (stress above 0.24 MPa at 50% strain), and easy recyclability.	[132]
	Pectin hydrogel from mandarin peel-based metalorganic frameworks composite	The pectin hydrogel from mandarin peel-based metalorganic frameworks composite was successfully examined for adsorptive removal of both Cr(VI)/Pb(II) ions from aqueous samples at pH 5.0 and 1.0, respectively, and proved to be useful as an adsorbent for toxic heavy metal elimination from wastewater.	[133]
	**Glycemic control**		
	Cardio-protective effects of pectin-insulin patch in streptozotocin-induced diabetic rats	The use of the pectin-insulin matrix patches (82.9 μg/kg) resulted in decreased blood glucose concentration and diabetes-induced disturbances in the lipid profile. Diabetes evoked an increase in MAP, which was attenuated in patch (82.9 μg/kg)-treated animals and decreased heart-to-body weight ratio, as well as cardiotropin-1, TNFα and hisCRP concentration.	[134]
	Agar and pectin on gastric emptying and post-prandial glycemic profiles	The gastric emptying and post-prandial glycemic profiles were examined for ten healthy male volunteers with three different test meals (450 kcal/500 mL): (i) a fiber-free meal; (ii) a meal with 2.0 g agar; (iii) a meal with 5.2 g pectin. The participants went through a [^13^C]-acetate breath test, along with serial blood sampling each time, and it was observed that agar and pectin delayed gastric emptying but have no impact on the post-prandial glucose response.	[135]
	**Encapsulating agent**		
	Microencapsulation of a-tocopherol with pectin and sodium alginate	The encapsulation efficiency of α-tocopherol in microencapsules formed using sodium alginate 1.5% *w*/*v* was 52.91% and pectin 2.0% *w*/*v*. α-Tocopherol microencapsules gave an encapsulation efficiency of 55.97% with the encapsulator, and 52.11% with the syringe method.	[136]
	Microencapsulation of curcumin in crosslinked jelly using fig pectin	Microencapsulation of curcumin in 0.75 *w*/*w*% jelly fig pectin was carried out by the vacuum spray drying (VSD) technique with 80–90 °C inlet temperature and 0.01 mPa pressure. The best encapsulation efficiency was observed with the VSD technique, and yield and loading efficiency was up to 91.56 ± 0.80%, 70.02 ± 1.96% and 5.45 ± 0.14%, respectively.	[137]
	**Therapeutic and pharmaceutical uses**		
	Pectin with anti-constipation activity	The pectin extracted from the roots of *Arctium lappa* L. with dosages of 200 mg/kg and 400 mg/kg exhibited strong anti-constipation activity in vivo. The *Arctium lappa* L. pectin-treated groups perhaps had improved small intestinal movement rate, and had significantly increased weight of feces in constipated mice.	[138]
	Pectin–honey hydrogel enhances wound healing	The wound area reduction rate was faster in rats treated with the pectin–honey hydrogel, liquid Manuka honey and pectin hydrogel compared to the control group, was significantly faster in the pectin–honey hydrogel group; unexpectedly, the pectin hydrogel displayed more rapid wound healing than the liquid Manuka honey.	[21]
	Natural film based on pectin and allantoin for wound healing	The pectin–allantoin films comprise two well-differentiated layers, one-layer rich in allantoin (regenerative layer), and one rich in pectin as an antimicrobial and protective layer to the wound. An in vivo assay illustrated a notable decrease of time period in the wound healing process by approximately 25%.	[139]
	PectaSol-C-modified citrus pectin, an inhibitor of galectin-3	Approximately 41% increased cell proliferation, 36% decreased caspase-3 activity and 33.6% increased substrate-dependent adhesion was observed in the presence of rhGal-3 compared to the control case (*p* < 0.001). Treatment of cells with a non-effective dose of PTX (100 nM) and 0.1% PectaSol-C-modified citrus pectin in combination revealed synergistic cytotoxic effects, with 75% reduced cell viability and a subsequent 3.9-fold increase in caspase-3 activity.	[140]
	Apple pectin as an adjunct to irinotecan therapy of colorectal cancer	The novel enzymatically extracted apple pectin reduced the viability of HCT 116 and Caco-2 colorectal cancer cells, induced apoptosis and increased intracellular reactive oxygen species production. Furthermore, enzymatically extracted apple pectin enhanced the cytotoxic and proapoptotic effect of irinotecan (at concentrations below its IC_50_), and exhibited potent anti-inflammatory properties.	[141]
	Modified citrus pectin prevents blood–brain barrier disruption in mouse subarachnoid haemorrhage by inhibiting galectin-3	Four micrograms of MCP attenuated post-SAH blood–brain barrier disruption and galectin-3 upregulation in brain capillary endothelial cells. Coimmunoprecipitation assay confirmed physical interactions between galectin-3 and TLR (toll-like receptor) 4. R-galectin-3 blocked the neuroprotective effects of MCP.	[142]

## Data Availability

Not applicable.
